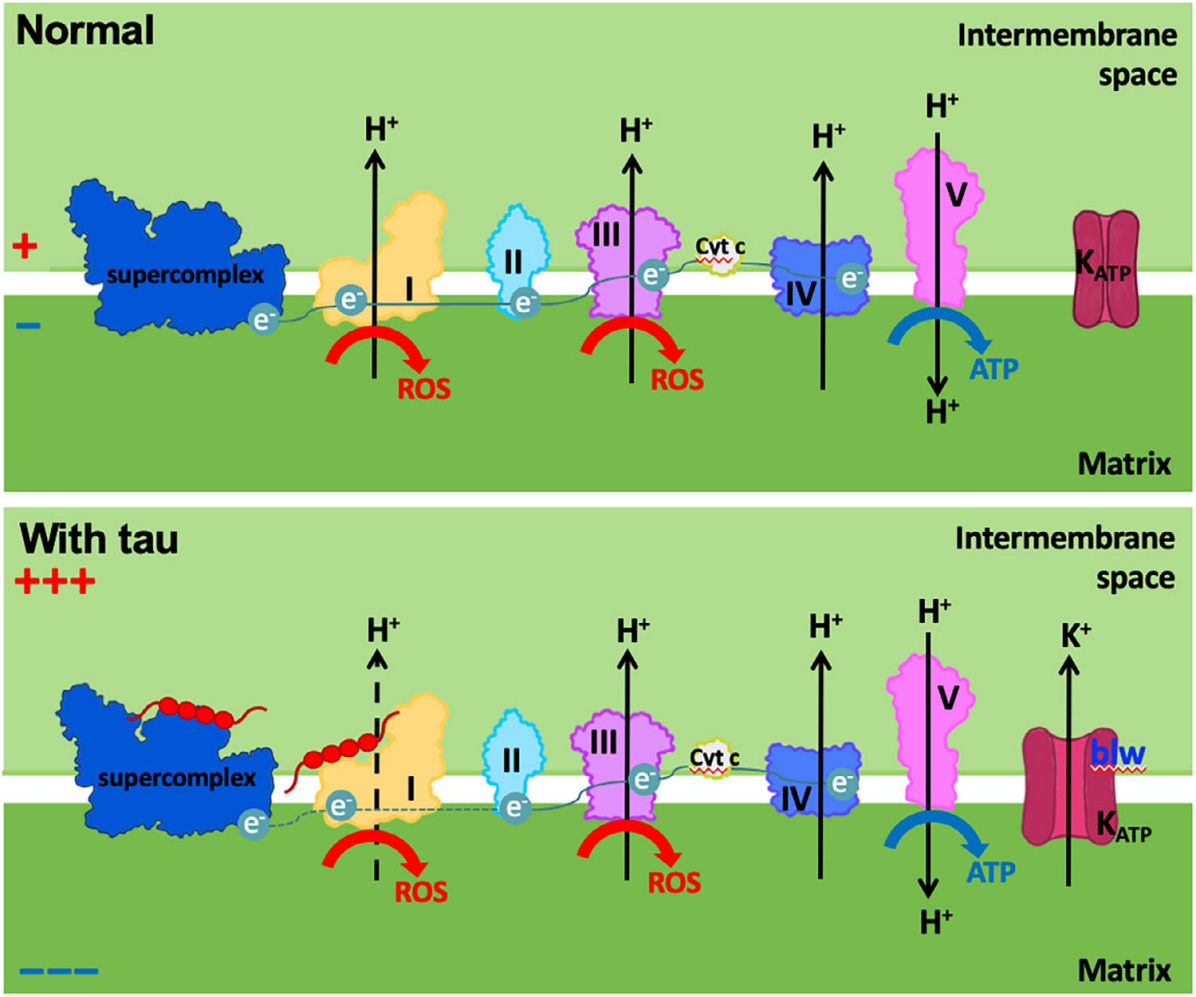# Tau disrupts OXPHOS complexes and hyperpolarizes mitochondria

**DOI:** 10.1002/alz70855_100654

**Published:** 2025-12-23

**Authors:** Arisa Tamura, Marie Noguchi, Taro Saito, Akiko Asada, Kanae Ando

**Affiliations:** ^1^ Tokyo Metropolitan University, Minami osawa, Japan; ^2^ Tokyo Metropolitan University, Hachioji, Tokyo, Japan

## Abstract

**Background:**

The abnormal accumulation of the microtubule‐binding protein tau is a pathological feature in a group of neurodegenerative diseases called tauopathy. Tau accumulation is thought to cause neuronal death, while underlying mechanisms are not fully understood. In cellular and animal models and data from patients suffering from tauopathy, various mitochondrial abnormalities have been reported. In this study, we aimed to elucidate the causal relationships among these abnormalities and their contribution to tau‐induced neurodegeneration.

**Method:**

In *Drosophila*, human tau expression causes neurodegeneration without the formation of neurofibrillary tangles. Tau proteins exist mainly in detergent‐soluble form with phosphorylation at disease‐related sites, suggesting that this model recapitulates the early stage of tau abnormality and toxicity of soluble tau. We used this model to analyze the effects of tau on ATP levels and the activity of OXPHOS complexes biochemically. Mitochondrial membrane potential and ROS levels were analyzed by imaging. mRNA levels were analyzed by qRT‐PCR. To analyze neurodegeneration, vacuole areas in the optic lobes were quantified with paraffin sections.

**Result:**

We found that tau expression reduces ATP levels and increases oxidative stress in the brain. Mitochondrial membrane potential was elevated, and the activities of OXPHOS complex I (CI) and those of supercomplexes were reduced. mRNA expression of OXPHOS genes was upregulated, and mitochondrial quantity was similar, suggesting that lowered CI activity is not due to a reduction in mitochondrial quantity or gene expression but disruption of CI at the protein level.

To investigate the causal relationships of these mitochondrial changes in tau‐induced neurodegeneration, we analyzed the effects of the co‐expression of *bellwether* (*blw*), encoding the alpha subunit of complex V, with tau. Co‐expression of *blw* suppressed tau‐induced photoreceptor degeneration. *blw* co‐expression did not restore ATP levels or lower ROS in tau‐expressing flies, but corrected hyperpolarization and increased CI activity.

**Conclusion:**

Our results indicate that tau causes mitochondrial hyperpolarization and lowers CI activity *in vivo*. Since suppression of mitochondrial hyperpolarization correlates with increased CI activity and suppression of neurodegeneration, targeting mitochondrial hyperpolarization may be a novel strategy to mitigate tau toxicity.